# Utilizing Genotype Imputation for the Augmentation of Sequence Data

**DOI:** 10.1371/journal.pone.0011018

**Published:** 2010-06-08

**Authors:** Brooke L. Fridley, Gregory Jenkins, Matthew E. Deyo-Svendsen, Scott Hebbring, Robert Freimuth

**Affiliations:** 1 Department of Health Sciences Research, Mayo Clinic, Rochester, Minnesota, United States of America; 2 Department of Molecular Pharmacology and Experimental Therapeutics, Mayo Clinic, Rochester, Minnesota, United States of America; Erasmus University Medical Center, Netherlands

## Abstract

**Background:**

In recent years, capabilities for genotyping large sets of single nucleotide polymorphisms (SNPs) has increased considerably with the ability to genotype over 1 million SNP markers across the genome. This advancement in technology has led to an increase in the number of genome-wide association studies (GWAS) for various complex traits. These GWAS have resulted in the implication of over 1500 SNPs associated with disease traits. However, the SNPs identified from these GWAS are not necessarily the functional variants. Therefore, the next phase in GWAS will involve the refining of these putative loci.

**Methodology:**

A next step for GWAS would be to catalog all variants, especially rarer variants, within the detected loci, followed by the association analysis of the detected variants with the disease trait. However, sequencing a locus in a large number of subjects is still relatively expensive. A more cost effective approach would be to sequence a portion of the individuals, followed by the application of genotype imputation methods for imputing markers in the remaining individuals. A potentially attractive alternative option would be to impute based on the 1000 Genomes Project; however, this has the drawbacks of using a reference population that does not necessarily match the disease status and LD pattern of the study population. We explored a variety of approaches for carrying out the imputation using a reference panel consisting of sequence data for a fraction of the study participants using data from both a candidate gene sequencing study and the 1000 Genomes Project.

**Conclusions:**

Imputation of genetic variation based on a proportion of sequenced samples is feasible. Our results indicate the following sequencing study design guidelines which take advantage of the recent advances in genotype imputation methodology: Select the largest and most diverse reference panel for sequencing and genotype as many “anchor” markers as possible.

## Introduction

In the last five years, the capabilities and technology for genotyping large sets of single nucleotide polymorphisms (SNPs) has increased significantly. Current genome-wide SNP arrays have the capability to genotype over one million SNP markers across the genome. This advancement in technology has led to an increased number of completed and on-going genome-wide association studies (GWAS) for various complex disease and drug-related phenotypes. These GWAS have resulted in more than 350 publications and over 1500 SNPs implicated for association with multiple (>80) disease phenotypes or traits [1]. However, the SNPs identified are not necessarily the functional variant and many GWAS studies are moving into the next phase of disease mapping involving the validation, augmentation and refining of these putative regions or loci [2]. The task of determining the “causative” variant(s) is difficult since 43% of associated SNPs are located in intergenic regions, and 45% are located within intronic regions of known genes [1].

Indirect association, as a result of linkage disequilibrium (LD), is a key factor in the success of genetic association studies. As a result of LD, a disease-susceptibility SNP need not be genotyped, as long as it is “tagged” by a SNP or set of SNPs that are genotyped (i.e., SNPs in LD with the disease-susceptibility SNP are genotyped). Recently this concept has been further exploited by the introduction of methods to impute genotypes at untyped markers, based on genotypes at typed markers and information about LD within the region [3], [4], [5], [6], [7], [8], [9], [10], [11], [12]. These methods are particularly useful in the context of failed genotyping and combining data across multiple platforms and recently have been extended to untyped markers using a reference data set [8], [10], [11].

One approach for following up replicated findings from a GWAS would be to determine all genetic variation within the locus, especially rarer variants not currently included on GWAS SNP arrays, as they may play an important role in the etiology of the disease [13]. This could be accomplished using the 1000 Genomes Project. However, one limitation of the use of 1000 Genomes Project for imputation of markers in a locus of interest is that the possible “deleterious” or “protective” alleles may not be represented in this relatively “healthy” cohort. An alternative approach would be to catalog all variants by sequencing the locus in the study subjects [14], followed by association analysis of each variant in the locus. However, sequencing is still relatively expensive and it may be cost prohibitive to sequence a region on a large set of individuals. A more cost effective approach would be to sequence a portion of the individuals, possibly selected based on the distribution of the phenotype and/or haplotypes, and then employ genotype imputation methods [15], [16], [17], [18] for imputing the sequenced markers in the remaining individuals. This approach could also be augmented with the additional inclusion of data from the 1000 Genomes Project.

In this manuscript we explore the use of the recently developed genotype imputation method implemented in MACH [16] for sequencing studies with the goal of localizing possible functional variants through statistical analysis. In doing so, we explore a variety of approaches for carrying out the imputation of untyped markers using a reference panel consisting of sequencing data for a fraction of the study participants. The various approaches are implemented using data from a candidate gene sequencing study conducted at the Mayo Clinic and data from the 1000 Genomes Project (http://www.1000genomes.org) [19].

## Materials and Methods

### Mayo Sequencing Study: *GENE1*

To explore various approaches for imputation of untyped markers using a reference panel determined from sequencing data, we utilized a recently completed sequencing study for a gene which we will denote as *GENE1* (unpublished data). Little is known in regard to common genetic variations within *GENE1*, and even less is known in regard to the relationship of *GENE1* genotypes with protein function or clinical phenotypes. In an attempt to address these issues, we have sequenced *GENE1* in 288 samples from the Coriell Institute's publically available “Human Variation Panel” (http://ccr.coriell.org/Sections/Collections/NIGMS/Populations.aspx?PgId=177&coll=GM), consisting of 96 African American (AA), 96 Caucasian American (CA), and 96 Han Chinese American (HCA). DNA was extracted by the Coriell Institute from EBV transformed lymphoblastoid cell lines.

As a consequence of *GENE1* being a relatively short gene (5 kb), the entire gene was sequenced using Sanger sequencing technology. PCR primers were designed to span the entire gene plus 1 kb beyond the 5′ and 3′ UTR. PCR products were bi-directionally sequenced on an Applied Biosystems 3730 DNA Analyzer and analyzed with SoftGenetic's Mutation Surveyor software. Sixty variants were cataloged across the three racial groups; eleven of these variants were previously-known SNPs that have entries in dbSNP (http://www.ncbi.nlm.nih.gov/projects/SNP/). **Table 1** presents a summary of the 31 polymorphisms that are either contained in HapMap (www.hapmap.org) or have a minor allele frequency (MAF)>1%.

**Table 1:** Summary of sequence data for *GENE1* for variants with MAF>1% or in HapMap.

		African American		White non-Hispanic American		Han Chinese American	
Marker	Position	ObsHET	MAF	ObsHET	MAF	ObsHET	MAF
1*	1270	0.385	0.203	0.415	0.303	0.474	0.416
5*	1541	0.365	0.182	0.427	0.307	0.479	0.417
6	1753	0	0	0.021	0.01	0	0
8	1811	0.021	0.01	0.031	0.026	0	0
9*	1812	0.062	0.031	0.104	0.062	0.26	0.193
10	1962	0	0	0.021	0.01	0	0
11*	1968	0	0	0	0	0.135	0.068
16	2829	0.021	0.01	0	0	0	0
17	3092	0	0	0	0	0.021	0.01
18	3145	0	0	0	0	0.021	0.01
19	3150	0.083	0.052	0	0	0	0
21	3456	0.521	0.396	0.417	0.312	0.469	0.411
22	3525	0	0	0	0	0.052	0.036
24	4399	0.115	0.057	0	0	0.146	0.083
25*	4467	0.052	0.026	0	0	0.146	0.083
27	4893	0.042	0.021	0	0	0	0
28*	5016	0.01	0.005	0	0	0	0
29*	5031	0	0	0.052	0.026	0	0
33*	5523	0.053	0.026	0.083	0.042	0.26	0.193
37	5974	0.021	0.011	0.042	0.021	0	0
39*	6166	0.385	0.203	0.469	0.432	0.365	0.224
40	6237	0.021	0.01	0	0	0	0
41	6265	0	0	0	0	0.062	0.031
42*	6311	0.01	0.005	0	0	0	0
44	6862	0	0	0.052	0.026	0	0
45	7036	0.031	0.016	0	0	0	0
48	7262	0	0	0	0	0.073	0.057
57*	7975	0.031	0.016	0	0	0	0
58	8057	0.021	0.01	0.094	0.047	0.011	0.005
59	8187	0.021	0.01	0	0	0	0
60	8230	0.021	0.01	0	0	0	0

*SNP Marker in HapMap; used as typed genotypes in all samples (i.e., markers on a GWAS SNP array).

MAF = minor allele frequency based on imputed “dosage” or expected genotype, position = physical base-pair location of the SNP based on build 36, ObsHET = observed heterozygote rate.

### 1000 Genomes Project: *COMT*

The initial goal of the 1000 Genomes Project was to sequence the entire genome in approximately 1200 individuals as a means of documenting all human genetic variation. Recently, the number of samples to be sequenced has increased to over 2000 samples [20]. As of September 20, 2009 (release 2009_04), there were 172 HapMap samples sequenced from three racial groups (57 U.S. residents with northern and western European ancestry (CEU), 56 Yoruba people of Ibadan, Nigeria (YRI) and 59 individuals from the Tokyo, Japan and Beijing, China (JPNCHB)) available for download and analysis from the 1000 Genomes Project. The data was part of a Pilot Project, in which samples were sequenced at, on average, 2x–4x coverage using ABI's SOLiD, Roche's 454 and Illumina's Solexa sequencing technologies. This pilot study was completed by the 1000 Genomes Consortium to evaluate the use of LD information and sequence data from multiple samples to aid in the genotype calling from low coverage, whole genome sequencing (http://www.genome.gov/26524516). Once downloaded, we focused on the region of the genome where the gene *COMT* is located based on the March 2006 build of the human genome as shown on the UCSC genome browser at http://genome.ucsc.edu/ (chromosome 22, 18243040–18336530). Four-hundred and six SNP markers were determined to be in the CEU population, 517 SNP markers in the YRI population and 290 SNP markers in the JPTCHB population.

### Genotype Imputation using Sequencing Data

To explore various approaches for imputing untyped markers to augment sequence data, using a reference panel determined from sequencing a portion of the study participants, we will utilize sequence data available for *GENE1* and *COMT*. To assess the various approaches, we have created the following hypothetical experiment. A SNP marker within a gene (e.g., *GENE1* or *COMT*) has been determined to be associated with a disease phenotype based on a large GWAS, in which all subjects have been genotyped for a set of SNPs on a large SNP array (e.g., Affymetrix Genome-Wide Human SNP Array 6.0 or Illumina human1M-duo array). The GWAS consists of subjects from three different racial groups. To follow-up the association findings, denser genotyping of the gene using sequencing technologies (Sanger or Next-Generation) was completed in a proportion of the individuals from each racial group. Based on the sequence data for the subset of the study participants, we wish to impute untyped markers for the remaining subjects using current genotype imputation methods. Based on previous reports regarding comparison of genotype imputation methods [21], [22], [23], we have elected to use MACH for the imputation [16].

In assessing the use of sequence data for genotype imputation, the experiment varied: the proportion of samples sequenced (or the size of the reference data), the number of markers genotyped for all subjects (“anchor” SNPs) and how these markers were selected, imputation based on the sequenced participants (“reference panel”) unphased genotypes or most-likely phased haplotypes and imputation based on race specific reference haplotypes or all reference haplotypes (regardless of race). The various simulation scenarios investigated using *GENE1* from the Mayo Study and *COMT* from the 1000 Genomes Project are outlined in the following sections.

To assess the accuracy of the various simulation scenarios, we *a priori* determined the samples to be considered the reference panel, using all of their sequencing data. For the rest of the sample considered the “observed” sample, non-anchor SNPs were masked, but retained and considered to be the “true” genotypes. These “true” genotypes could then be compared to the imputed markers arrived at using MACH's most likely genotype call, with anchor SNPs in common between the reference population and “observed” sample. In addition to computing the concordance rates based on most likely genotype call, we also used the –mask option in MACH to compute quality measures for the imputed markers and estimated MAF based on the expected genotype (i.e., dosage).

### Imputation Scenarios for *GENE1*

**Table 2** displays the various genotype imputation scenarios investigated for use with sequence data. The first simulation scenario (Scenario 1) was one in which the reference haplotypes for the sequenced samples was determined using the most likely haplotypes produced by fastPHASE [24]. The eleven prior known SNP markers were treated as the “anchor” markers genotyped on all subjects with the remaining markers sequenced in a portion of the subjects. The haplotype estimation and the genotype imputation were both completed by race.

**Table 2:** Imputation Scenario Design Summary.

Factors Varied for Imputation		Scenario			
		1	2	3	4
**Use of Phased Reference Haplotypes for Imputation**					
Most likely phase reference haplotypes		**X**	**X**		**X**
Unphased genotypes				**X**	
**Reference Haplotypes**					
Race specific reference haplotypes		**X**			**X**
All reference haplotypes, regardless of race			**X**		
**Number of Markers Genotyped on All Subjects**					
*GENE1*	Three				**X**
	Five				**X**
	Seven				**X**
	Ten	**X**	**X**	**X**	
*COMT*	Tag SNPs	**X**	**X**	**X**	
	SNPs on GWAS array				**X**

In Scenario 2, imputation was completed by race (as for Scenario 1), but imputation was based on all reference haplotypes for all three races. To assess the variation in haplotype assignment and impact on imputation accuracy, imputation Scenario 3 was completed, by race, with only unphased genotypes for the sequenced samples (i.e., no phased reference haplotypes used). It should be noted that not all genotype imputation methods allow for the use of only unphased genotypes and may require reference haplotypes. Lastly, Scenario 4 assessed the impact of the number of “anchor” markers genotyped for all subjects, with either 3 markers (Scenario 4.3), 5 markers (Scenario 4.5), or 7 markers (Scenario 4.7) used in the imputation with the same design as Scenario 1.

### Imputation Scenarios for *COMT*

For the *COMT* study, based on data from the 1000 Genomes Project, the “anchor” markers were selected for two situations: a candidate gene study (Scenarios 1, 2 and 3) and a genome-wide association study (Scenario 4) (**Table 2**). Scenarios 1, 2 and 3 were similar to the corresponding scenarios for *GENE1*. For scenario 4 the *COMT* and *GENE1* scenarios differed in implementation. Scenario 4 for *GENE1* was used to assess the impact of varying the number of anchor markers (either 3, 5 or 7), while for *COMT* this scenario assessed the impact of how the “anchor” markers were selected: one based on candidate gene study in which SNPs were selected by tagging the region and one based on *a priori* defined SNPs on a GWAS SNP array. SNPs were defined to be on the GWAS panel if they were contained in HapMap, as many large GWAS SNP arrays are designed based on HapMap SNPs.

To mimic the candidate gene scenario, we consider the common situation where the anchor markers, genotyped on all subjects, would be LD based tag SNPs. Therefore, to determine anchor markers for *COMT*, tagging was completed by race using Haploview v4.1 [25] with a minimum inclusion minor allele frequency (MAF) of 0.05 and a r^2^ of 0.8. This resulted in 125, 187 and 117 tagSNPs selected for CEU, YRI, and JPNCHB populations, respectively. These markers, for each race, were then taken to be the “anchor” markers genotyped on all samples in the candidate gene study. To mimic the case in which the “anchor” markers were on a genome-wide SNP array (Scenario 4), SNPs were chosen as any SNPs present in HapMap, resulting in 25 for the CEU population, 50 SNPs in the JPTCHB population and 32 SNPs in the YRI population [26].

## Results

### Genotype Imputation for *GENE1*

Sequencing of *GENE1*, using Sanger Sequencing technology, detected five novel variants with MAF greater than 5% and 20 novel variants with MAF greater than 1% not previously reported (**Table 1**). None of the SNP genotypes deviated from Hardy-Weinberg equilibrium, where HWE was tested by race using Haploview [25]. By using our study population consisting of Caucasian Americans (CA), African Americans (AA) and Han-Chinese Americans (HCA), we are able to ensure the comparability of LD structure used in the imputation. This may not be true if the 1000 Genomes Project data were to be used, as the population of interested may not be represented. The various scenarios for genotype imputation using sequence data for polymorphic markers in gene *GENE1* was completed using MACH 1.0 (http://www.sph.umich.edu/csg/abecasis/mach/index.html) using the commands: mach1 -d markers.dat -p pedigree.ped -h hap.haplos -s all.snp --rounds 150 --greedy --mask 0.02 --geno --dosage --quality -o machOut for scenarios using reference haplotypes and mach1 -d markers.dat -p pedigree.ped --rounds 150 --greedy --mask 0.02 --geno --dosage --quality -o machOut for the scenarios in which only unphased genotypes were used in the imputation (Scenario 3). Percent of concordant genotype calls was computed by comparing the imputed genotypes to the “true” observed genotypes.

**Figure 1:**
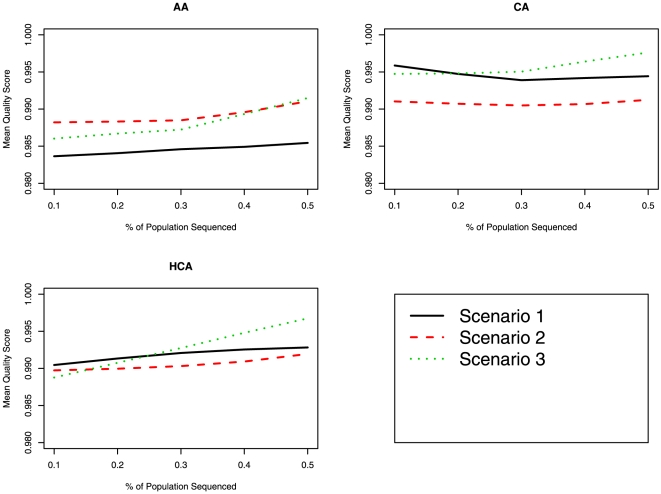
Comparison of mean SNP imputation quality score between the various imputation scenarios for *GENE1*. The proportion of the sample used as the reference panel is displayed on the X-axis and the mean SNP imputation quality score is displayed on the Y-axis.

**Figure 2:**
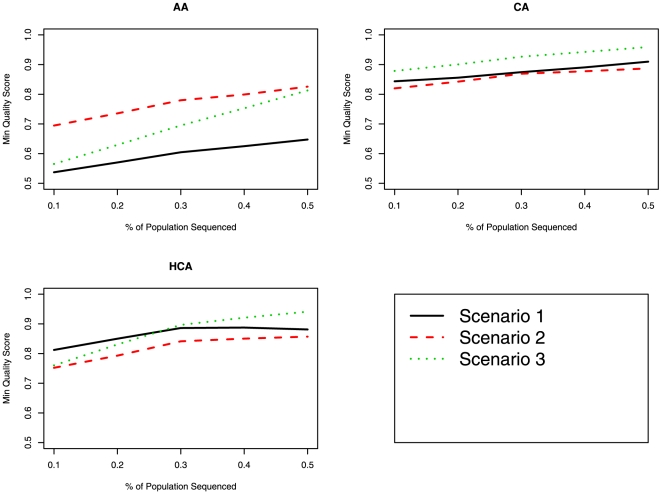
Comparison of minimum SNP imputation quality score between the various imputation scenarios for *GENE1*. The proportion of the sample used as the reference panel is displayed on the X-axis and minimum SNP imputation quality score is displayed on the Y-axis.

**Table 3** displays the concordance rates for Scenarios 1, 2, and 3, while **Figures 1** and **2** display the mean and minimum SNP quality score, as provided by MACH (based on expected imputed genotype), for the three races and three scenarios. In the AA and HCA groups, using only race specific reference haplotypes (Scenario 1) produced the lowest mean quality scores and concordance for imputation. This observation agrees with other publications, which state that a more diverse set of reference haplotypes increases the accuracy in imputation [27]. For imputation of CA, the results were similar between the three methods for imputation, with Scenarios 1 and 2 producing the best concordance and Scenarios 1 and 3 producing the highest mean and minimum quality score. In terms of the impact of the number of “anchor” markers on imputation, the greater the number of markers, the higher the quality and concordance in imputation. **Table 4** shows the concordance as a function of number of “anchor” markers when 50% of the samples where sequenced; the same trend was observed when a smaller proportion of samples were sequenced and used as the reference (data not shown).

**Table 3:** Concordance values between the “true” genotype and most likely imputed genotype for *GENE1* for Scenarios 1, 2 and 3.

		Proportion Sequenced				
Scenario	Race	0.1	0.2	0.3	0.4	0.5
1	AA	0.962	0.965	0.973	0.962	0.974
	CA	0.988	0.991	0.968	0.989	0.964
	HCA	0.963	0.973	0.969	0.964	0.97
2	AA	0.975	0.971	0.975	0.972	0.971
	CA	0.985	0.978	0.978	0.976	0.977
	HCA	0.98	0.975	0.976	0.975	0.978
3	AA	0.969	0.971	0.973	0.975	0.97
	CA	0.977	0.972	0.972	0.971	0.972
	HCA	0.968	0.972	0.972	0.972	0.972

**Table 4:** Concordance values between the “true” genotype and most likely imputed genotype for *GENE1* for various number of “anchor” markers.

	Number of Anchor Markers			
Race	3	5	7	11
AA	0.966	0.972	0.973	0.974
CA	0.952	0.960	0.962	0.964
HCA	0.958	0.962	0.972	0.970

Table presents results for scenario with 50% of the samples sequenced.

Lastly, in general, as the proportion (or number) of samples sequenced increased (i.e., larger set of reference haplotypes), the quality of the imputation increased. Thus, not only does number of markers genotyped on all subject impact the accuracy of imputation, so does the size of the reference sample. In addition to variation in results due to the number of markers genotyped on all subjects and size of the reference set, two different reference sets of the same size (i.e., different samples selected for sequencing) resulted in slightly different imputation accuracy (data not shown).

### Genotype Imputation with 1000 Genomes Project

The various scenarios for genotype imputation using the 1000 Genomes Project sequence data for polymorphic markers in *COMT* was completed using MACH 1.0 using the commands using the commands: mach1 -d markers.dat -p pedigree.ped -h hap.haplos -s all.snp --rounds 150 --greedy --mask 0.02 --geno --quality -o machOut for all scenarios.

**Figure 3:**
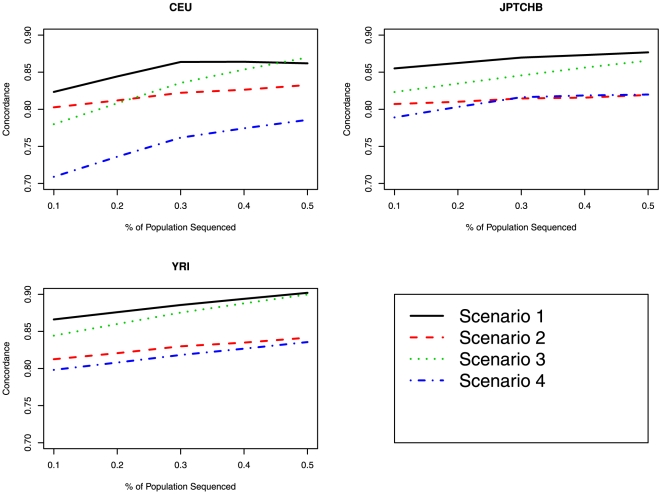
Comparison of concordance rates between the various imputation scenarios for *COMT*. The proportion of the sample used as the reference panel is displayed on the X-axis and the percent concordant between the “true” genotype and the imputed most likely genotype is displayed on the Y-axis.

**Figure 4:**
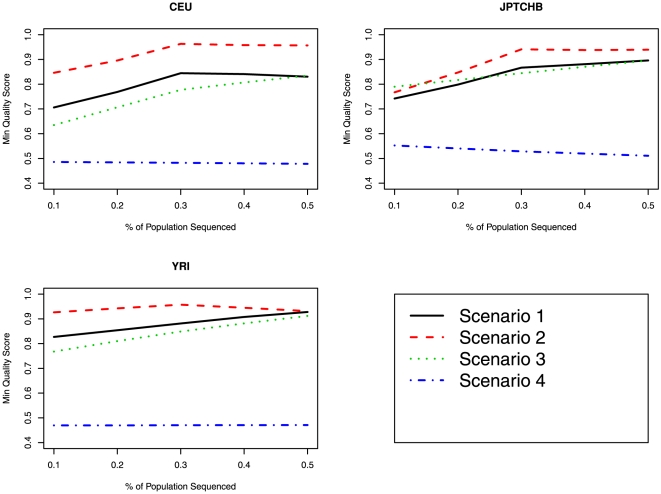
Comparison of minimum SNP imputation quality score between the various imputation scenarios for *COMT*. The proportion of the sample used as the reference panel is displayed on the X-axis and the minimum SNP imputation quality score is displayed on the Y-axis.

As completed for *GENE1*, we looked at the SNP concordance rates and SNP quality score for *COMT* for the various imputation scenarios (**Figures 3** **and** **4**). Figures 3 and 4 display the relationship between the concordance and minimum quality score for the four scenarios in relation to the proportion of the population sequenced. In terms of highest minimum quality score, imputation based on reference haplotypes for all races (Scenario 2) was the “best”; however in terms of concordance between the observed and imputed most likely genotype, imputation based on race specific reference haplotypes was “best” (Scenario 1). The range (minimum – maximum) in mean concordance rates for Scenario 1 was 0.82–0.87 for CEU, 0.86–0.88 for JPTCHB and 0.87–0.92 for YRI.

The figures and table show that anchor SNPs based on a tag SNP approach dramatically outperformed the approach where anchor SNPs were based on a large SNP array (Scenario 4), in terms of both concordance rates and minimum SNP quality scores. In terms of highest minimum quality score, imputation based on reference haplotypes for all races (Scenario 2) was the “best”; however in terms of concordance between the observed and imputed most likely genotype, imputation based on race specific reference haplotypes was “best” (Scenario 1). The figures also show a slight increase in imputation performance as the size of the reference panel increased.

**Figure 5:**
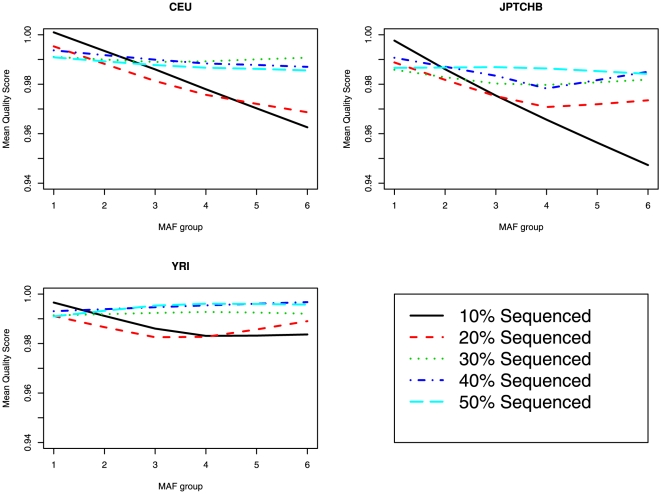
Comparison of mean SNP imputation quality score versus MAF for *COMT* imputation scenario 1. The MAF (group 1: 0≤MAF≤0.05, group 2: 0.05<MAF≤0.10, group 3: 0.10<MAF≤0.20, group 4: 0.20<MAF≤0.30, group 5: 0.30<MAF≤0.40, group 6: 0.40<MAF≤0.50) is displayed on the X-axis and the mean SNP imputation quality score is displayed on the Y-axis.

Lastly, with one goal of sequencing to be to detect rare variants, we compared, for the three races, the relationship between the estimated MAF, based on the imputed dosage, after imputation and the quality of imputation (**Figure 5**). We observed the mean quality score decreased as the MAF increased and that as the proportion of samples sequenced increased, the average imputation accuracy improved for common variants.

## Discussion

In this manuscript present the use of the recently developed genotype imputation method for sequencing studies, where the reference panel consisting of sequencing data for a fraction of the study participants. By sequencing only a portion of the samples for the follow-up of signals detected from a GWAS, followed by imputation in the remaining samples, one can significantly reduce the cost to localize the punitive variant involved in the etiology of complex disease and pharmacogenomic phenotypes. In addition, by utilizing sequence data on a portion of individuals in the study, we are able to have a perfectly matched reference panel, in terms of linkage disequilibrium, without relying on the assumption that the HapMap populations represent our study population (as HapMap based haplotypes are the current standard reference data used for genotype imputation).

Sequencing a portion of our study population also allows us to determine and assess association of rare variants not present in the HapMap database. Upon completion of the 1000 Genomes project (http://www.1000genomes.org), incorporation of this information can also be utilized to determine variants not already identified in public databases. However, one limitation of the use of 1000 Genomes project for imputation for all subjects is the possibly that this “healthy” cohort does not adequately represent the genetic diversity observed in the affected individuals (i.e., individuals with the disease). Thus, there will still be a need to sequence individuals, in particular, those individuals with the disease or phenotype of interest.

Our results, based on a Sanger sequencing study of a candidate gene and preliminary data from the 1000 Genomes Project for *COMT*, show that imputation of untyped markers based on sequencing a portion of the study participants is a reasonable, cost-saving approach for disease mapping and refinement of putative regions detected with GWAS. However, there was not a clear scenario that was the “best” for genotype imputation across the two genes and three races. At the completion of the 1000 Genomes Project, future research will be needed to determine optimal approaches to incorporate this valuable information to inform future genetic association studies. In addition, further research is needed to develop cost effective sequencing study designs and analysis methods that incorporate the uncertainty in the reference haplotypes and imputation into the association analysis. Based on results from this study, we recommend a few guidelines in designing sequencing studies to take advantage of the recent advances in genotype imputation methodology:

1. Select the largest and most diverse reference panel for sequencing, with respect to both haplotypes and phenotype. One can also use sequencing data from the 1000 Genomes data in addition to the sequencing of a portion of the study participants (i.e., reference panel consists of data from sequencing a portion of individuals with the disease/phenotype of interest and data from the 1000 Genomes Project)
2. Given that sequencing produces unphased genotypes, if possible, imputation should be carried out on the unphased genotypes in the reference panel as opposed to the most likely phase haplotypes to account for the uncertainty in haplotype assignment.
3. Genotype as many “anchor” markers as possible, in that, the number of markers genotyped on all subjects impacts accuracy. Therefore, additional genotyping of a few common SNP markers not already genotyped on all subjects using a cost effective platform, like Taqman, may be needed if the GWAS SNP array does not provide adequate coverage in the locus to be sequenced.
